# Research Hotspots and Trends of Interventions for Sarcopenic Obesity: A Bibliometric Analysis

**DOI:** 10.7759/cureus.64687

**Published:** 2024-07-16

**Authors:** Ning Zhang, Jiarong He, Xuan Qu, Lin Kang

**Affiliations:** 1 Department of Geriatrics, Peking Union Medical College Hospital, Peking Union Medical College, Chinese Academy of Medical Sciences, Beijing, CHN; 2 Department of Hemodialysis, Gansu Provincial People's Hospital, Lanzhou, CHN

**Keywords:** nutrition, resistance exercise, sarcopenic obesity, multimodal intervention, bibliometric analyses

## Abstract

Sarcopenic obesity, characterized by both obesity and sarcopenia, significantly impacts health and independence of affected individuals. There is an urgent need to explore effective strategies for addressing or preventing sarcopenic obesity. An initial critical step is to promptly assess the impact of academic research in this field, considering factors such as geographical regions, authors, journals, and institutions. It is also essential to analyze current trends and identify potential areas that may inspire future researchers to conduct further studies, ultimately improving public health outcomes for individuals with sarcopenic obesity. To achieve this, bibliometric research was conducted using the Web of Science Core Collection database to identify English language articles and reviews focusing on sarcopenic obesity interventions published between January 1, 2004, and June 15, 2024, followed by a literature review.

A total of 929 English-language articles were collected, consisting of 645 research articles and 284 reviews. Research output in the field has shown significant growth since 2017, reaching a peak of 139 papers in 2022. The United States leads in publication output with 234 papers and a total of 13,971 citations, highlighting substantial international collaboration. Both the United States and Europe are recognized as key academic hubs for sarcopenic obesity intervention research, characterized by robust academic interactions. Moreover, there has been a notable increase in publication volume from China, South Korea, and Japan. Noteworthy authors in this field include Boirie Y from Université Clermont Auvergne in France, Prado CM from the University of Alberta in Canada, Cruz-Jentoft AJ from Hospital Universitario Ramon y Cajal in Spain, and Prado CM from the University of Alberta, known for their high citation count. The University of Alberta leads in the number of publications, while the University of Verona in Italy leads in citation frequency. Journals with higher publication volumes in sarcopenic obesity intervention include Nutrients, Clinical Nutrition, and Journal of Cachexia Sarcopenia and Muscle. Among the top 20 keywords, the most relevant interventions for sarcopenic obesity are exercise, nutrition, resistance training, physical activity, and muscle strength. The primary evidence currently available suggests that resistance training is the most effective method for enhancing muscle strength in sarcopenic obesity patients. Additionally, combining protein supplementation with resistance exercise has shown encouraging results in reducing fat mass in these individuals. To progress in this field, it is crucial to foster collaboration among countries, regions, and academic institutions, promoting multidisciplinary partnerships.

## Introduction and background

The prevalence of sarcopenic obesity (SO), a high-risk geriatric syndrome characterized by the combination of obesity and age-related loss of muscle mass and strength, is increasing among adults aged 65 years and older. This subset of the population is at risk of synergistic complications from both sarcopenia and obesity [1]. SO is a newly recognized form of obesity prevalent among the elderly, with projections indicating a substantial impact on 100 to 200 million individuals globally over the next 35 years [2]. Variations in prevalence rates are observed across different countries due to disparities in defining criteria, with factors such as gender, race, and age playing key roles. Considering diverse definitions and study cohorts, the estimated global prevalence of SO ranges from 5% to 10% [3]. Interestingly, the prevalence rates do not significantly differ between genders, although higher rates are reported among Hispanics compared to non-Hispanic blacks, particularly in individuals aged 80 years and above [4]. Population-representative data in the United States revealed a prevalence rate of SO of 28.3% among individuals aged over 60 years, with a notably higher prevalence of 66.6% observed in Mexican Americans [5]. Notably, advanced age is identified as a risk factor for the development of SO. Despite limited research on SO prevalence in the elderly population in China, a study involving 948 community-dwelling elderly individuals from West China Hospital of Sichuan University revealed an overall prevalence of 6.0%, with rates of 7.3% in males and 4.3% in females [6].

Existing researches have demonstrated that the presence of SO is correlated with a variety of adverse clinical outcomes. These include metabolic issues like dyslipidemia, diabetes mellitus, metabolic syndrome, insulin resistance, and decreased levels of vitamin D. Furthermore, this condition is linked to geriatric syndromes such as cognitive impairment, functional limitations, increased risk of falls, depressive symptoms, dementia, frailty, osteoporosis, short sleep duration, low physical activity levels, fatigue, and disability. Additionally, SO can impact cancer outcomes and treatment by reducing overall, recurrence-free, and disease-free survival rates, increasing surgical complications and hospital length of stay, and decreasing tolerance to therapy. It also raises the risk of mortality from various causes, including cardiovascular disease, heart failure, and cardiovascular surgery, as well as morbidity outcomes like hypertension, lung diseases, stroke, and arthritis. Moreover, SO is associated with the development of other clinical conditions like hospitalization, poor nutritional status, limited improvement in activities of daily living, dysphagia after stroke, reduced quality of life, inflammation, and poor recovery in knee flexion range of motion after total knee replacement [7,8].

The impact of sarcopenic obesity on health outcomes, particularly among the elderly, has garnered growing interest. However, there is a noticeable gap in bibliometric research that specifically examines intervention strategies for this condition. This study employs the "Bibliometrix" package within the R environment to dissect the current research landscape on interventions for sarcopenic obesity. Additionally, a thorough literature review was conducted in this area. The findings of this study are poised to offer valuable insights for guiding future research endeavors in this domain.

## Review

Ethics, data sources, and search strategies

**Figure 1:**
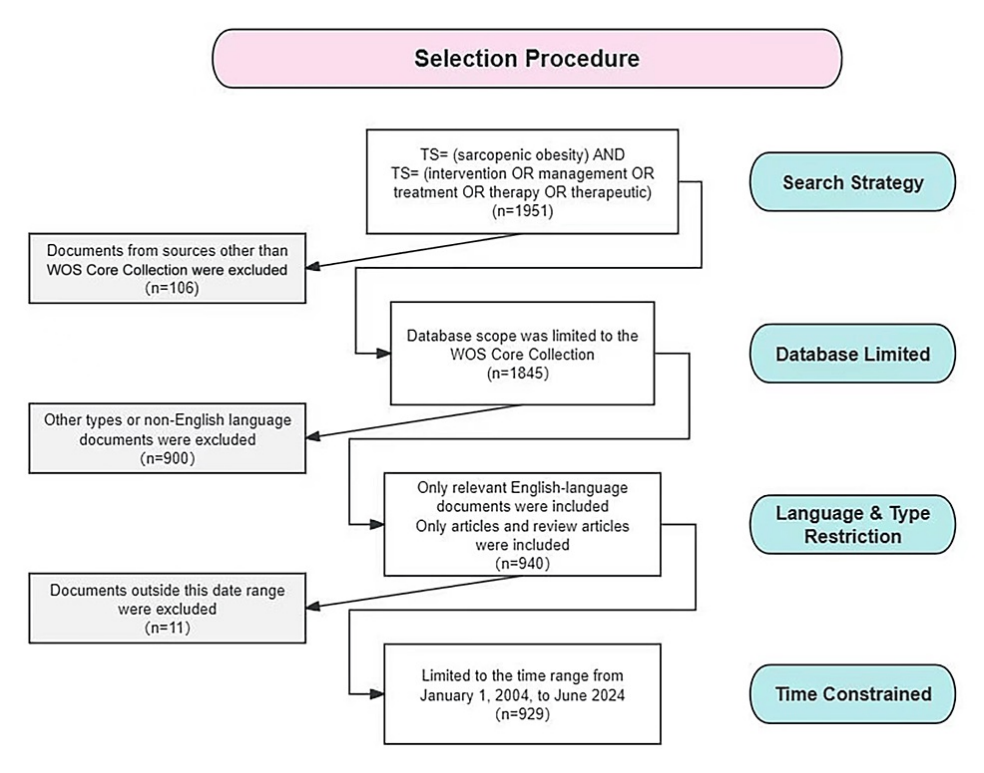
Flow diagram of the study selection procedure. WOS: Web of Science; TS: Topic Search

The study was reviewed and approved by the Ethics Committee of Peking Union Medical College Hospital (approval number: I-23PJ738). The literature data for this study were retrieved from the Web of Science Core Collection database, which includes academic publications from over 250 scientific disciplines globally [9]. Researchers have validated the reliability of this database for bibliometric analyses. The bibliometric analysis for this study covers the period from January 1, 2004, to June 15, 2024. The search query used is as follows: Topic Search (TS)=(sarcopenic obesity) AND TS=(intervention OR management OR treatment OR therapy OR therapeutic). Only relevant English documents were included, with letters, comments, and meeting abstracts excluded. A total of 929 documents from 65 countries/regions were found, including 645 articles and 284 reviews involving contributions from 5110 authors, 1,607 institutions, and 402 journals. A detailed overview of the study selection process is shown in Figure 1, following the Preferred Reporting Items for Systematic Reviews and Meta-Analyses (PRISMA) flow diagram.

Data analysis

The screened and optimized raw dataset was exported in TXT file format after confirming the accuracy of the data. This dataset contains key information such as titles, authors, keywords, institutions, countries/regions, citations, journals, and publication dates. Microsoft Excel 2021 (Redmond, WA: Microsoft Corp.), VOSviewer (version 1.6.18) (Leiden, Netherlands: Centre for Science and Technology Studies {CWTS}, Leiden University), CiteSpace (version 6.1.R6) (Philadelphia, PA: Chaomei Chen, Drexel University), and the R package "Bibliometrix" (Milan, Italy: Massimo Aria, University of Milano-Bicocca) were utilized as the primary tools for data analysis and visualization. VOSviewer, developed by Nees Jan van Eck and colleagues in 2010, is a widely used graphical tool for extracting and analyzing key information from numerous publications [10]. It enables the exploration of collaborative relationships among countries/regions, authors, institutions, and keyword co-occurrences. On the other hand, CiteSpace, created by Chaomei Chen, generates network maps for specific research domains, unveiling critical insights such as potential trends, frontier hotspots, and research directions. This study utilized CiteSpace to perform co-occurrence and cluster analysis of information related to authors, research institutions, and countries [11]. Furthermore, Bibliometrix, developed by Aria and Cuccurullo in 2017, was utilized to analyze the evolution trends of keywords in the literature. It offers comprehensive bibliometric and scientometric analysis capabilities through the R language.

Publication and citation analysis

**Figure 2:**
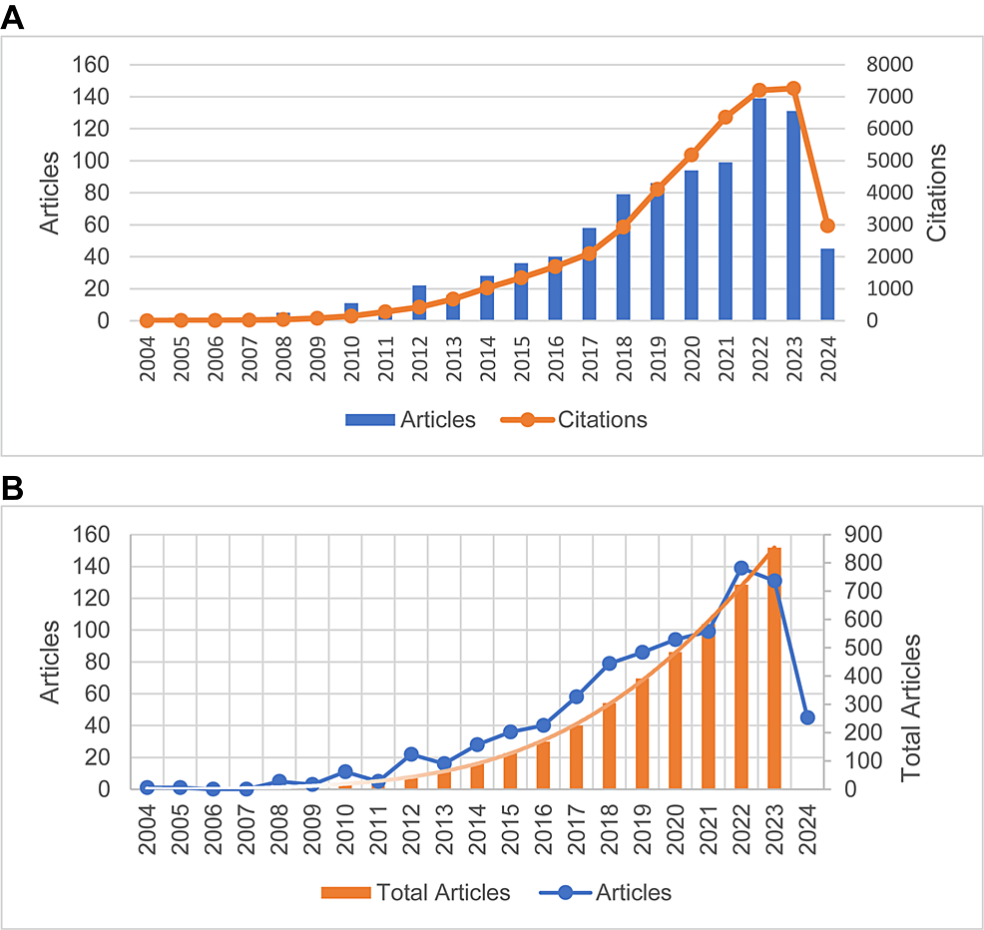
Trends in the published articles and citation counts of sarcopenic obesity intervention research from 2004 to 2024. (A) The annual publication quantity and citation frequency of research on sarcopenic obesity intervention from 2004 to 2024. (B) The annual publication quantity, cumulative publication quantity, and their polynomial fitting curves for sarcopenic obesity intervention from 2004 to 2024.

Figure 2A illustrates the progression of both publications and citations from 2004 to 2024. The data reveals a consistent increase in the number of annual publications and citations over the years. Initially, the publication count experienced fluctuations with lower numbers before 2014. However, a notable shift occurred in 2017, leading to a substantial rise in publications, peaking at 139 papers in 2022. On the other hand, the citation count displayed a more steady growth, reaching its peak of 7258 citations in 2023. It is worth noting that the data for 2024 is incomplete in the graph as data collection ended in mid-June. Figure 2B depicts a polynomial fit of the cumulative annual publication count (represented by the equation: y = -0.0007x⁵ + 0.0346x⁴ - 0.4499x³ + 3.1043x² - 8.2898x + 7.5714), with a high goodness of fit R² = 0.9998. This fitting curve clearly demonstrates an upward trajectory, suggesting ongoing rapid advancements in frontier research within this field.

Countries/regions analysis

Conducting a bibliometric analysis of the countries/regions from which publications originate helps us understand the geographical distribution of research in this field and identify the key areas of focus. This approach also sheds light on the collaborative relationships between different countries/regions globally. Leading the research on sarcopenic obesity interventions, the United States and Italy stand out (Table 1). The United States takes the lead in both the number of publications (234 papers) and citations (13,971 times), surpassing Italy, which ranks second with 110 papers and 13,803 citations. This underscores the significant research capacity of the United States in this area. Furthermore, the contributions of France (12,515 citations), the United Kingdom (12,461 citations), and Germany (12,378 citations) are also noteworthy. Progress in a scientific field is not solely dependent on the efforts of one country or region but rather a result of collaborative contributions from multiple countries/regions.

**Table 1:** Ranking of the top 10 major countries/regions of sarcopenic obesity intervention from 2004 to 2024.

Rank	Countries	No. of documents	Countries	Total link strength	Countries	No. of citations
1	USA	234	Italy	219	USA	13,971
2	Italy	110	USA	202	Italy	13,803
3	China	92	France	202	France	12,515
4	South Korea	74	Germany	198	England	12,461
5	Germany	73	Spain	161	Germany	12,378
6	Japan	67	Sweden	150	Switzerland	11,272
7	England	62	Netherlands	145	Sweden	11,154
8	France	61	Israel	143	Spain	10,262
9	Canada	57	England	142	Belgium	10,010
10	Spain	55	Switzerland	131	Czech Republic	8423

**Figure 3:**
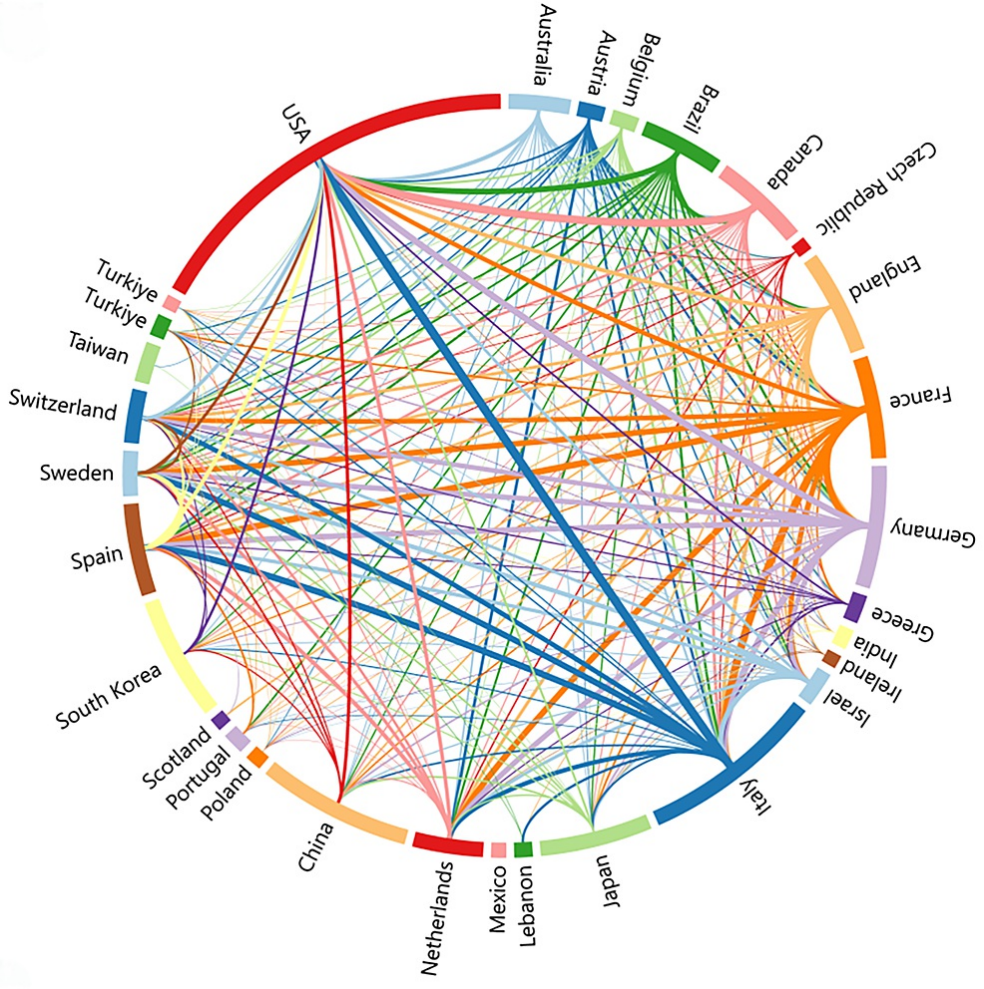
National/regional collaborative network mapping of sarcopenic obesity intervention from 2004 to 2024. Countries and regions clustering analysis was conducted using collaboration information data sourced from the Web of Science Core Collection (WoSCC) and visualized using Charticulator (Redmond, WA: Microsoft Research). The length of the outer circle corresponds to the number of documents issued by each country/region, while the connections within the circle depict cooperative relations between them. Lines of the same color as the respective country/region indicate the initiation of cooperation.

Using VOSviewer, we conducted a comprehensive analysis of the top countries/regions based on publication count. The collaborative relationships among these entities are visually represented in Figure 3 using a chord diagram. Each country/region is represented by a distinct colored band, with the width of the band indicating the extent of collaboration. The largest band in red represents the United States, closely followed by Italy, underscoring their significant contributions to sarcopenic obesity intervention research. Germany, France, China, Canada, and South Korea also make notable contributions. Particularly noteworthy are the strong academic connections among Italy, the United States, France, and Germany, evident from the thicker bands connecting them. Italy is particularly distinguished for its extensive and consistent academic collaborations with other nations, similar to France and Germany. On the contrary, while the United States engages in numerous collaborations, the intensity of its collaborative efforts appears slightly lower compared to the aforementioned European countries. Countries like Mexico, with fewer connections, tend to prioritize collaborations within their own borders.

**Figure 4:**
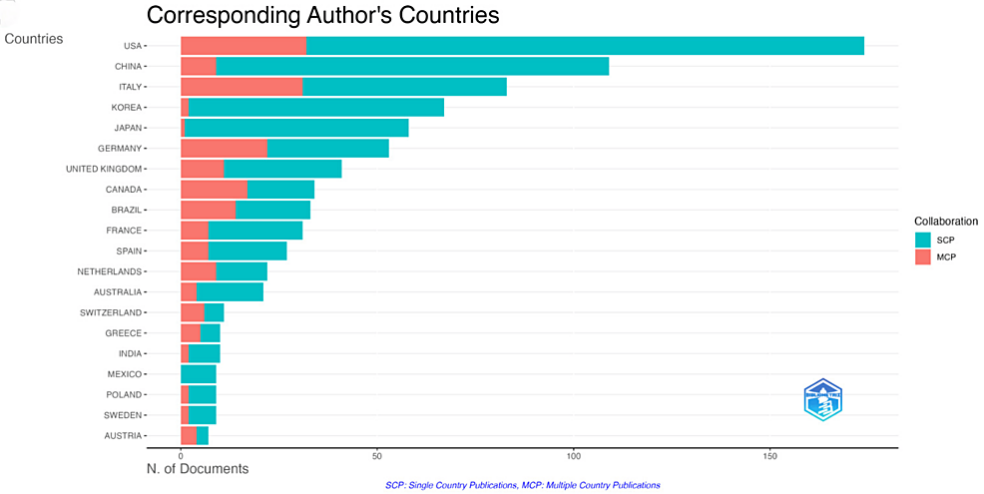
National collaborative network mapping of sarcopenic obesity intervention from 2004 to 2024. The proportion of single-country publications to multiple-country publications among the top 20 countries in terms of publication output indirectly reflects the academic collaboration tendencies of these countries.

Figure 4 illustrates the contributions of major countries/regions in sarcopenic obesity intervention research. The United States leads the chart, followed by China, Italy, South Korea, and Japan. Notably, the United States and Italy prioritize international academic partnerships. Canada and Australia have more internationally co-authored publications than domestic ones. In contrast, China, South Korea, and Japan emphasize domestic collaborations. Mexico stands out for its lack of international academic exchange in this field. This visualization highlights geographical research distribution and diverse collaborative behaviors among countries and regions. It shows the prevalence of international collaborations in Western countries versus the focus on domestic partnerships in East Asia, reflecting distinct scientific research approaches.

Author analysis

Table 2 displays the top 10 authors based on publication volume and citation count within the realm of sarcopenic obesity intervention research. The most prolific authors are Boirie Y from Université Clermont Auvergne in France and Prado CM from the University of Alberta in Canada, both with 16 publications. Other noteworthy authors include Kemmler W from the University of Nuremberg E in Germany and Batsis JA from the University of North Carolina Chapel Hill in the United States, each with over 10 publications. These authors hail from diverse countries, indicating a lack of regional concentration. Cruz-Jentoft AJ from Hospital Universitario Ramon y Cajal in Spain (558 citations) and Prado CM from the University of Alberta (462 citations) are the top two most cited authors, followed by Baumgartner RN from the University of Louisville (355 citations) and Batsis JA from the University of North Carolina Chapel Hill (352 citations). These high citation counts demonstrate the substantial impact and recognition of their research within the academic community.

**Table 2:** Ranking of the top 10 major authors of sarcopenic obesity intervention from 2004 to 2024.

Rank	Author	No. of documents	Total link strength	Countries/regions	Institution	Author	No. of co- citations	Total link strength	Countries/regions	Institution
1	Boirie Y	16	67	France	Universite Clermont Auvergne	Cruz-Jentoft AJ	558	7251	Spain	Hospital Universitario Ramon y Cajal
2	Prado CM	16	24	Canada	University of Alberta	Prado CM	462	5199	Canada	University of Alberta
3	Kemmler W	13	46	Germany	University of Erlangen Nuremberg	Baumgartner RN	355	6093	USA	University of Louisville
4	Batsis JA	11	25	USA	University of North Carolina Chapel Hill	Batsis JA	352	6276	USA	University of North Carolina Chapel Hill
5	Barazzoni R	10	92	Italy	University of Trieste	Kemmler W	264	2142	Germany	University of Erlangen Nuremberg
6	El Ghoch M	9	38	Italy	University of Modena and Reggio Emilia	Villareal DT	231	4837	USA	Baylor College of Medicine
7	Itani L	9	38	Lebanon	Beirut Arab University	Janssen I	228	3659	USA	Rush University
8	Scott D	9	9	Australia	Deakin University	Stenholm S	182	3085	Finland	University of Turku
9	Baracos VE	8	10	Canada	University of Alberta	Zamboni M	171	2596	Italy	University of Verona
10	Busetto L	8	74	Italy	University of Padua	Martin L	157	1516	Belgium	University of Antwerp

**Figure 5:**
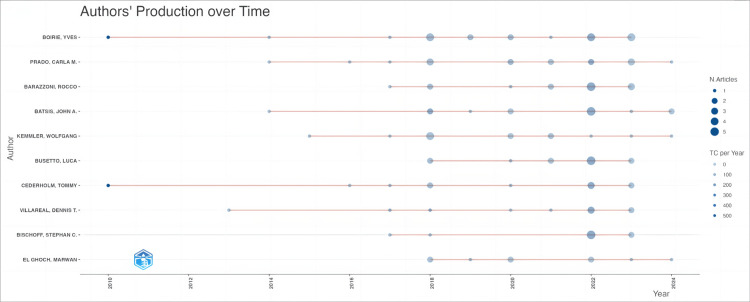
Network visualization of author publication volume, collaboration, and citation relationships in the field of sarcopenic obesity intervention from 2004 to 2024. The visualization analysis depicts the publications of the top ten authors ranked by frequency of occurrence from 2009 to 2024. In this visual analysis, the length of the line indicates the duration of sustained authorship, while the size of the dots indicates the number of publications in chronological order, and the color of the dots indicates the frequency of citations.

Figure 5 illustrates the publication activity of authors spanning from 2009 to 2024. The length of the lines along the horizontal axis signifies the duration of each author's active involvement in the field, while the size of the dots denotes the number of papers published in specific years. The color intensity of the dots indicates the frequency of citations. Boirie Y and Cederholm T have the lengthiest active periods, commencing their publications in 2010 and continuing to the present. Noteworthy is the spike in publication volume and citation counts in 2022, suggesting a significant breakthrough. Similar trends are noted in 2018 and 2023, albeit less prominently than in 2022. This hints at possible major advancements or discoveries in those years, leading to increased research output and academic recognition.

**Figure 6:**
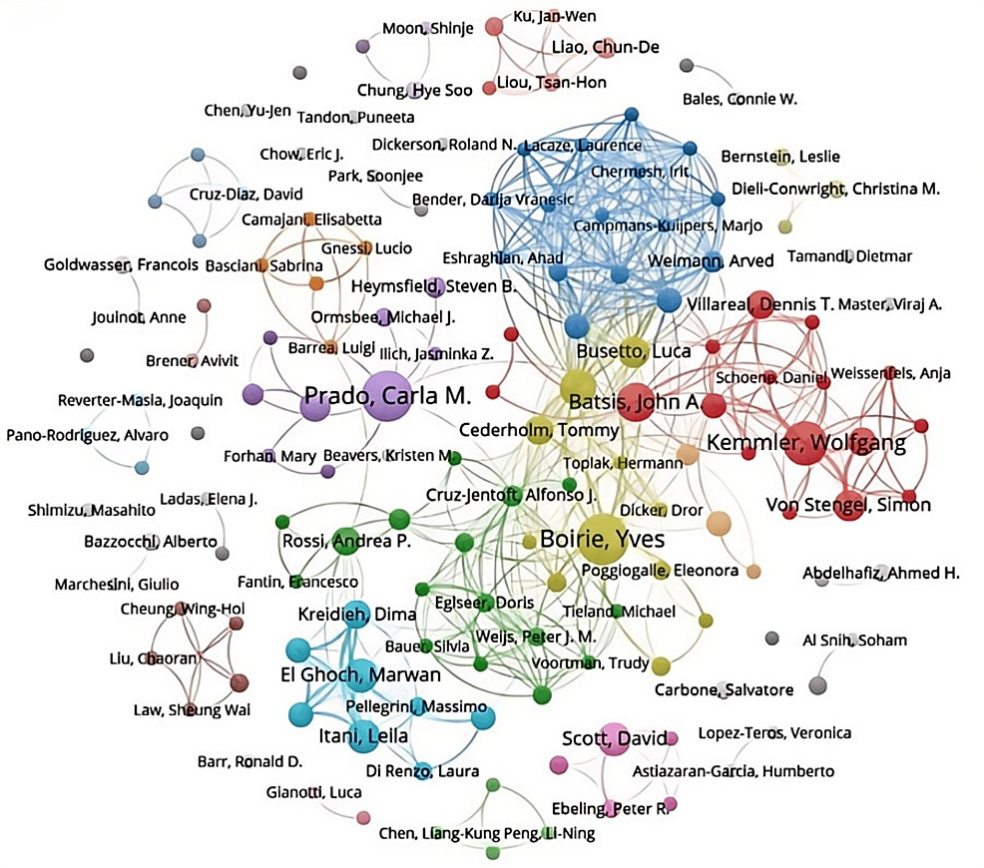
Network visualization of author publication volume, collaboration, and citation relationships in the field of sarcopenic obesity intervention from 2004 to 2024. The diagram displays co-occurring authors in sarcopenic obesity intervention research, with nodes colored to represent distinct author clusters. Node size indicates the frequency of co-occurrence, and links depict the relationships among co-occurring authors. All author information data in the figure are sourced from the Web of Science Core Collection (WoSCC) and visualized using VOSviewer.

**Figure 7:**
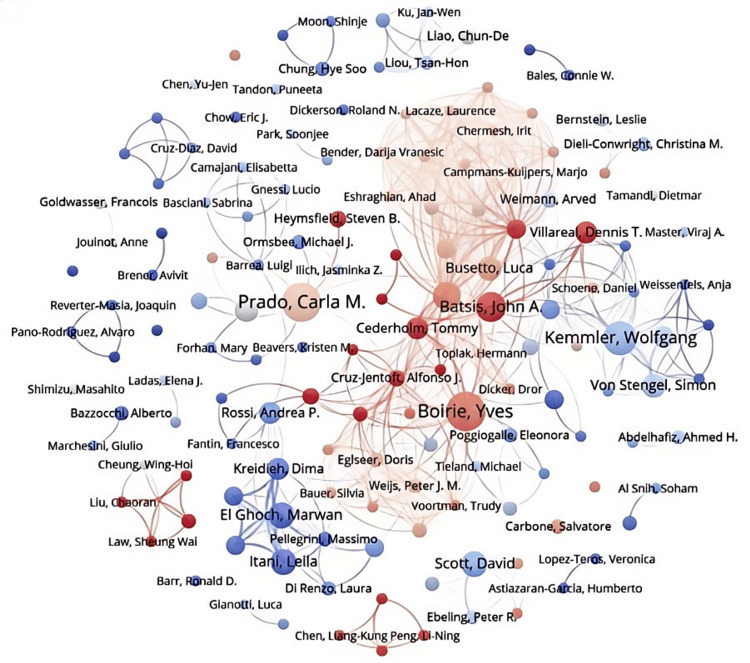
Network visualization of author publication volume, collaboration, and citation relationships in the field of sarcopenic obesity intervention from 2004 to 2024. The figure overlays the presentation of heat based on Figure 6, displaying the frequency of recent publication volume through different shades of color. All author information data in the figure are sourced from the Web of Science Core Collection (WoSCC) and visualized using VOSviewer.

Analyzing Figures 6, 7 provides a deeper insight into the collaborative dynamics among authors in the field. In Figure 6, authors are categorized into distinct clusters based on the frequency of their academic interactions. The prominent yellow cluster, centered around Boirie Y (the largest dot), includes closely connected authors like Busetto L, Cederholm T, and Dicker D. The upper blue cluster exhibits a more intricate network, featuring authors such as Weimann A, Eshraghian A, and Chermesh I. The red cluster on the right comprises authors like Batsis JA, Kemmler W, Villareal DT, and Von SS. Other notable clusters on the left side include the purple, light blue, and green clusters, involving authors like Prado CM, El Ghoch M, and Itani L. Importantly, these clusters often involve authors who are not geographically close, highlighting the significant role of international collaboration in advancing research on sarcopenic obesity interventions. Figure 7 complements Figure 6 by illustrating the strength of these collaborative ties more vividly. The intensity of the color indicates the frequency of interactions between authors, revealing that authors like Batsis JA, Cederholm T, and Cruz-Jentoft AJ have particularly close collaborations. Furthermore, a small cluster at the lower left corner demonstrates a strong collaboration between Liu C and Law SW, both based in China.

**Figure 8:**
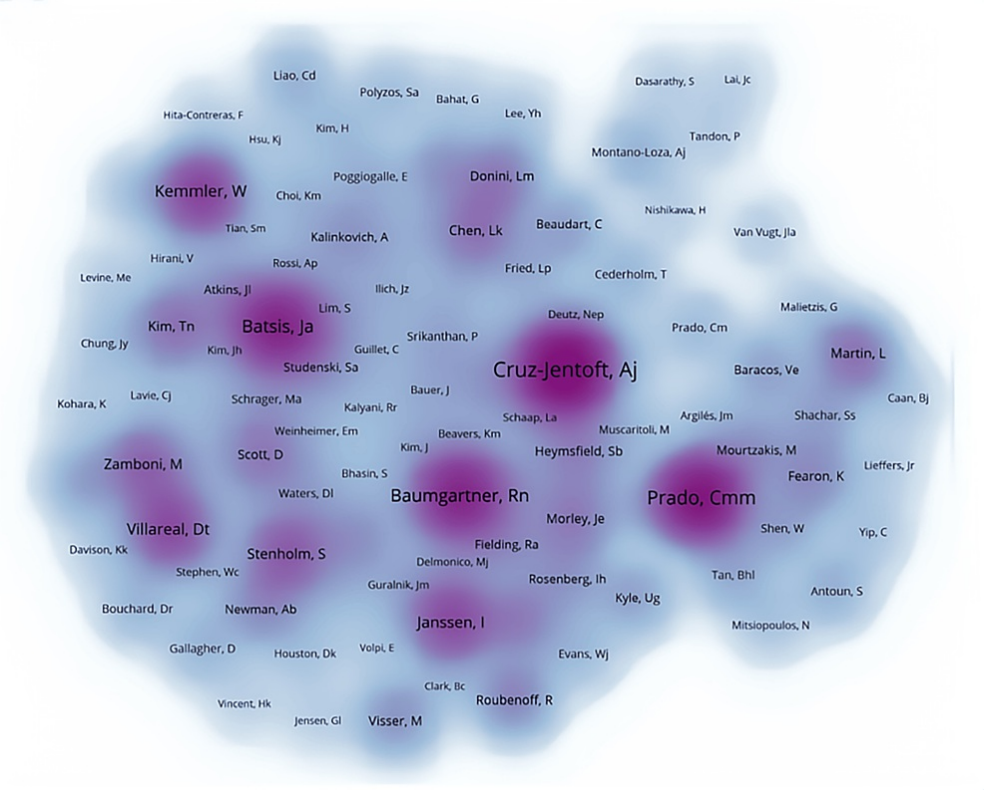
Network visualization of author publication volume, collaboration, and citation relationships in the field of sarcopenic obesity intervention from 2004 to 2024. The map overlays the authors’ publication volume, where the intensity of color depth indicates the volume of publications. All author information data in the figure are sourced from the Web of Science Core Collection (WoSCC) and visualized using VOSviewer.

Figure 8 provides a comprehensive overview of key authors in the field, showcasing their publication output and citation impact. The intensity of colors reflects the total number of publications, with darker shades indicating higher citation frequencies. Notably, authors like Cruz-Jentoft AJ, Prado CM, Baumgartner RN, and Batsis JA hold significant influence within the field, despite having weaker interconnections. Conversely, authors such as Zamboni M and Villareal DT, who also boast impressive citation counts, demonstrate stronger collaborative ties.

**Figure 9:**
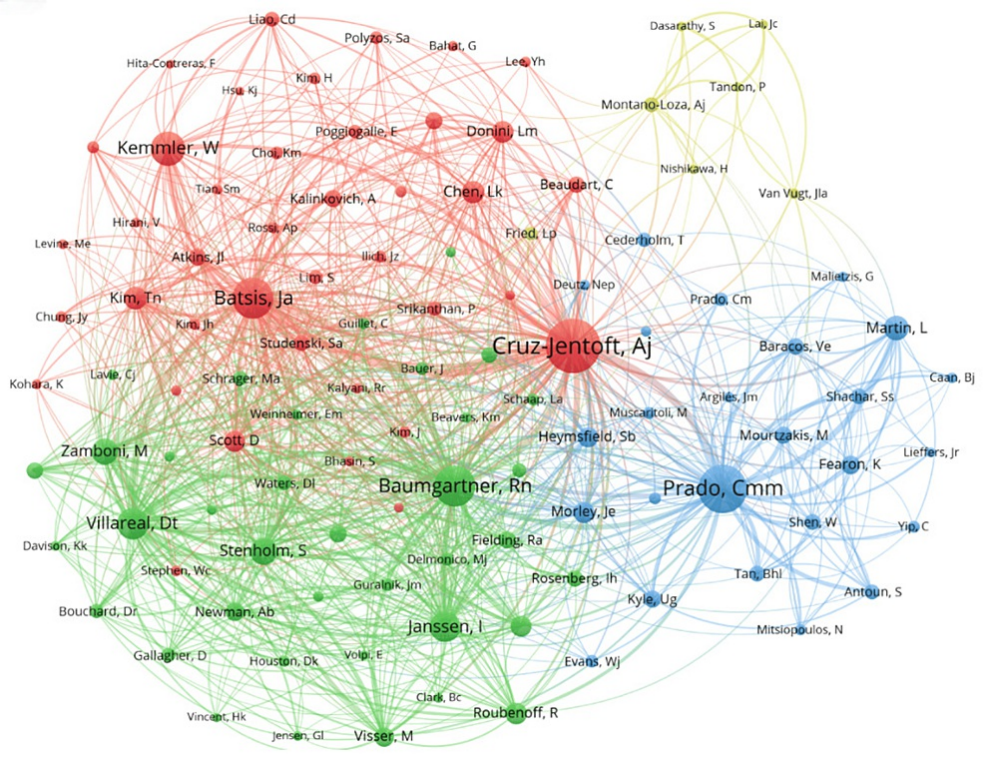
Network visualization of author publication volume, collaboration, and citation relationships in the field of sarcopenic obesity intervention from 2004 to 2024. The diagram illustrates co-cited authors in research on sarcopenic obesity intervention, with node size indicating citation frequency. This visualization, created using VOSviewer, succinctly captures and analyzes the interconnectedness of cited authors in this research area. All author information data in the figure are sourced from the Web of Science Core Collection (WoSCC) and visualized using VOSviewer.

Figure 9 illustrates the co-citation relationships among authors, where the thickness of the lines represents the frequency of co-citations, and the size of the dots indicates the frequency of co-citations. Co-citation refers to how often two authors are cited together in the same paper, revealing the relevance and similarity of their research. Based on this indirect citation relationship, authors in the field of sarcopenic obesity treatment are grouped into four main clusters. In the red cluster, authors with high co-citation frequencies such as Cruz-Jentoft AJ, Kemmler W, and Batsis JA hold prominent positions. This cluster includes authors mainly focused on geriatrics, internal medicine, and endocrinology, highlighting areas like geriatric medicine, public health, nutrition, and dietetics. The green cluster, encompassing authors like Baumgartner RN, Stenholm S, Janssen I, Villareal DT, and Zamboni M, focuses on related fields such as nutrition, internal medicine, endocrinology, and dietetics. These authors are primarily concerned with public health, geriatric medicine, and the dietary sciences. The blue cluster is centered around Prado CM, including authors like Fearon K and Martin L, among others. Their research spans nutrition, endocrinology and metabolism, biosocial science, and experimental medicine. Finally, the yellow cluster in the upper right includes authors such as Montano-Loza, Aldo J, and Tandon P, whose research mainly addresses gastroenterology, nutrition, and other scientific disciplines. In summary, the co-citation analysis visually maps the interconnections among key researchers in the sarcopenic obesity treatment field, highlighting the prominent researchers and their research foci within the domain.

Institution analysis

Table 3 highlights the top 10 institutions in sarcopenic obesity intervention research based on publication quantity and citation frequency. The University of Alberta in Canada leads with 35 publications, followed by the University of Clermont Auvergne in France (18 papers), Sapienza University of Rome in Italy (15 papers), and Verona University in Italy (14 papers). Notably, the University of Alberta demonstrates a significant advantage in publication quantity, indicating a strong commitment to this research area. In terms of citation frequency, Charles University in the Czech Republic ranks fifth with 8230 citations, with the top five most cited institutions including the University of Verona in Italy (9639 citations), Uppsala University in Sweden (8868 citations), and the University of Erlangen-Nuremberg in Germany (8551 citations).

**Table 3:** Ranking of the top 10 major institutions of sarcopenic obesity intervention from 2004 to 2024.

Rank	Institution	No. of publications	Original country	Institution	No. of citations	Original country
1	University of Alberta	35	Canada	University of Verona	9639	Italy
2	University of Clermont Auvergne	18	France	Uppsala University	8868	Sweden
3	Sapienza University of Rome	15	Italy	University of Erlangen-Nuremberg	8551	Germany
4	Verona University	14	Italy	Universita Cattolica del Sacro Cuore	8418	Italy
5	Friedrich Alexander Univ Erlangen Nürnberg	13	Germany	Charles University	8230	Czech Republic
6	Monash University	13	Australia	University of Alberta	4571	Canada
7	University of Melbourne	13	Australia	Johns Hopkins University	1363	USA
8	Tel Aviv University	12	Israel	Baylor College of Medicine	1169	USA
9	Universita Cattolica del Sacro Cuore	12	Italy	University of Clermont Auvergne	1028	France
10	University of Hohenheim	12	Germany	Dartmouth Hitchcock Medical Center	803	USA

**Figure 10:**
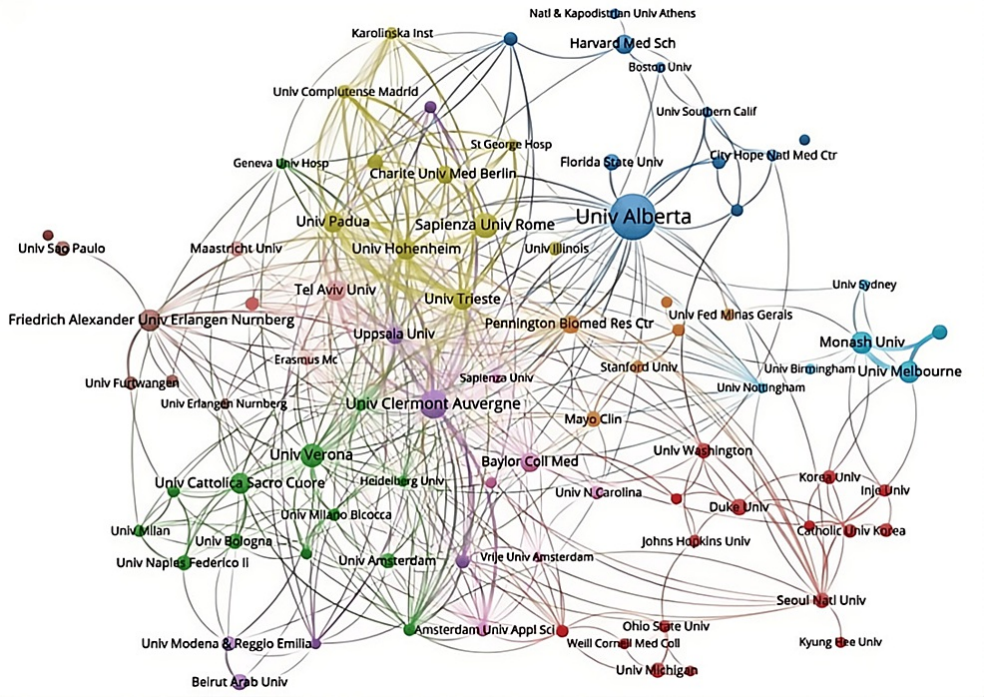
Institutional collaborative network mapping of sarcopenic obesity intervention research from 2004 to 2024. The graph illustrates the co-occurrence of research institutions, with node size representing the frequency of their occurrence together and connections indicating co-occurrence relationships. The size of each node indicates how frequently research institutions collaborate, while the links represent instances of their joint occurrences. All institution collaboration information data in the figure are sourced from the Web of Science Core Collection (WoSCC) and visualized using VOSviewer.

The collaboration networks among institutions were visualized and presented in Figure 10. The University of Alberta, which leads in publication quantity, is located in the blue cluster in the upper right corner. This cluster includes institutions mainly from North America, such as Florida State University and Harvard Medical School. The yellow cluster to the left contains Italian institutions like the University of Padua, the University of Trieste, and the Sapienza University of Rome, while the green cluster includes the University of Verona, the Universita Cattolica del Sacro Cuore, and the University of Milan. The red cluster on the right comprises institutions like Johns Hopkins University, Duke University, the University of Michigan, and the University of Washington. This analysis reveals that the different clusters represent collaboration groups with distinct geographical characteristics.

Journal analysis

**Figure 11:**
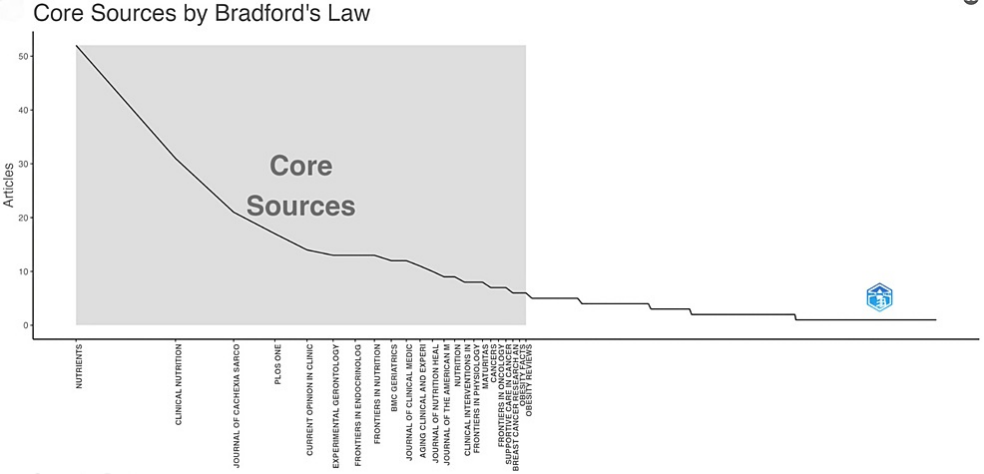
Network visualization of journal publication volume, collaboration, and citation relationships in the field of sarcopenic obesity intervention from 2004 to 2024. Bradford’s law can be applied to academic journals in the field, as evidenced by the grey-shaded area in the figure. This area encompasses the journals that have made significant contributions to the field, arranged in descending order based on the number of articles. The visualization overlays the publication volume of journals, with color intensity indicating the volume of publications. All journal citation information data in the figure are sourced from the Web of Science Core Collection (WoSCC) and visualized using VOSviewer.

Table 4 ranks high-impact journals in the field of sarcopenic obesity intervention based on publication volume and influence. The gray shaded area in Bradford's law, depicted in Figure 11, highlights journals that have made significant contributions to the field, listed in descending order of article count. Analyzing the aforementioned figure and table together, noteworthy journals with higher publication volumes include Nutrients (52 papers), Clinical Nutrition (31 papers), and Journal of Cachexia Sarcopenia and Muscle (21 papers). It is important to mention that all three of these journals are ranked in Q1 according to Journal Citation Reports (JCR). Furthermore, among the top 10 journals with the highest publication volumes in this field, nine are ranked Q2 and above, which is particularly significant in terms of citation frequency. All top 10 journals in terms of citations are ranked in Q2 and above, with seven in Q1. These journals include Clinical Nutrition (1416 citations), American Journal of Clinical Nutrition (1374 citations), Journals of Gerontology Series A: Biological Sciences and Medical Sciences (1208 citations), and Journal of the American Geriatrics Society (1021 citations). These findings suggest that research outcomes and advancements in sarcopenic obesity intervention garner considerable attention in the academic community.

**Table 4:** Ranking of the top 10 major journals of sarcopenic obesity intervention from 2004 to 2024.

Rank	Journal	No. of publications	Impact factor (Journal Citation Reports 2022)	JCR quartile	Co-cited journal	No. of citations	Impact factor (Journal Citation Reports 2022)	JCR quartile
1	Nutrients	52	5.9	Q1	Clinical Nutrition	1416	6.3	Q1
2	Clinical Nutrition	31	6.3	Q1	American Journal of Clinical Nutrition	1374	7.1	Q1
3	Journal of Cachexia Sarcopenia and Muscle	21	8.9	Q1	Journals of Gerontology Series A: Biological Sciences and Medical Sciences	1208	5.1	Q2
4	PLOS One	17	3.7	Q2	Journal of the American Geriatrics Society	1021	6.3	Q1
5	Current Opinion in Clinical Nutrition and Metabolic Care	14	3.1	Q3	Journal of Cachexia Sarcopenia and Muscle	955	8.9	Q1
6	Experimental Gerontology	13	3.9	Q2	PLOS One	818	3.7	Q2
7	Frontiers in Endocrinology	13	5.2	Q1	Nutrients	786	5.9	Q1
8	Frontiers in Nutrition	13	5.0	Q2	Journal of the American Medical Directors Association	783	7.6	Q1
9	BMC Geriatrics	12	4.1	Q2	Age and Ageing	733	6.7	Q1
10	Journal of Clinical Medicine	12	3.9	Q2	Journal of Applied Physiology	713	3.3	Q2

**Figure 12:**
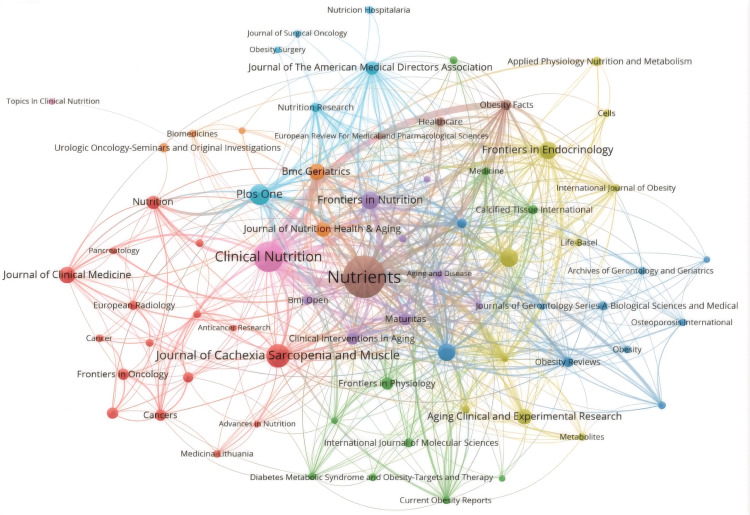
Network visualization of journal publication volume, collaboration, and citation relationships in the field of sarcopenic obesity intervention from 2004 to 2024. The graph depicts the co-citation relationships among research journals, with node size indicating the frequency of co-occurrence and connections indicating co-citation relationships. Node size reflects the significance and influence of journals in the network. All journal citation information data in the figure are sourced from the Web of Science Core Collection (WoSCC) and visualized using VOSviewer.

Figure 12 illustrates the co-citation relationships among various journals. The central circle in the figure represents the journal Nutrients, surrounded by journals like Obesity Facts and Healthcare, focusing on nutrition, endocrinology, and healthcare sciences. On the left, a red cluster emphasizes geriatrics, oncology, and medicine, with journals such as the Journal of Cachexia Sarcopenia and Muscle, Frontiers in Oncology, Journal of Clinical Medicine, and Nutrition. The light blue cluster above includes publications like PLOS One, Nutrition Research, and the Journal of the American Medical Directors Association, with research themes in nutrition, geriatrics, and multidisciplinary studies. Additionally, there are clusters in blue, yellow, green, and purple representing journals like Clinical Nutrition, Frontiers in Endocrinology, Frontiers in Physiology, and Frontiers in Nutrition.

**Figure 13:**
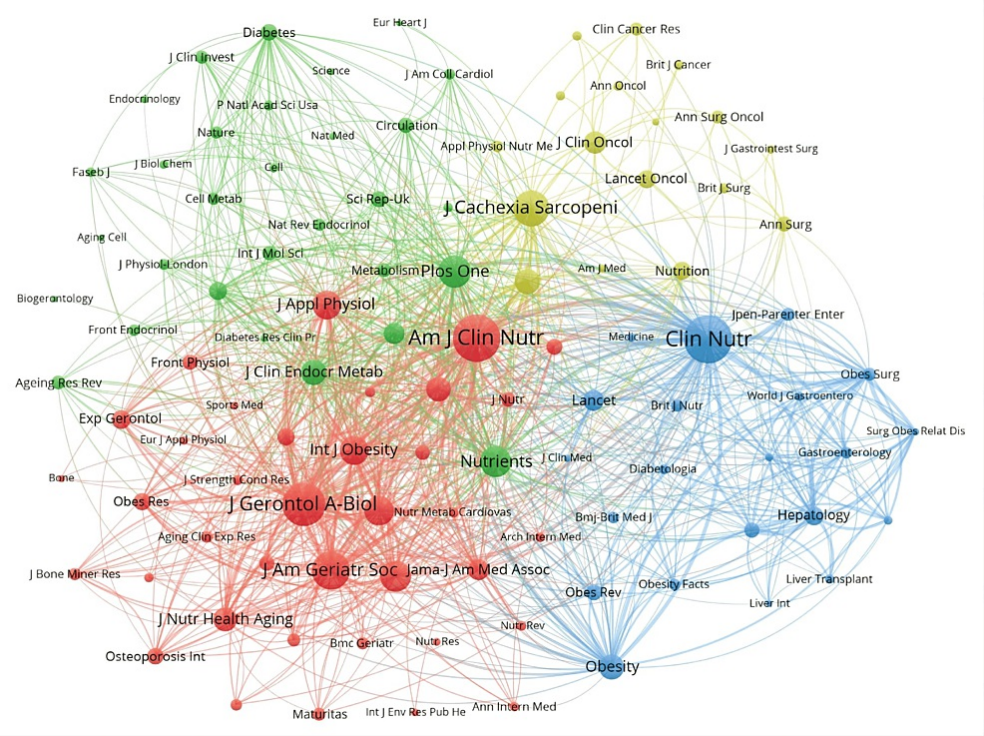
Network visualization of journal publication volume, collaboration, and citation relationships in the field of sarcopenic obesity intervention from 2004 to 2024. Visualization analysis of the collaboration network of journals in VOSviewer. Journals in different clusters are distinguished by nodes of different colors, with node size representing their frequency of occurrence. All journal citation information data in the figure are sourced from the Web of Science Core Collection (WoSCC) and visualized using VOSviewer.

Figure 13 illustrates the network of collaboration among major journals in the field, with clusters of different colors representing various relationships. The red cluster emerges as particularly influential, containing journals like Journals of Gerontology Series A: Biological Sciences and Medical Sciences, American Journal of Clinical Nutrition, Journal of the American Geriatrics Society, and Journal of the American Medical Directors Association, all centered around geriatrics, nutrition, and dietetics. The blue cluster, led by Clinical Nutrition, includes journals like Obesity and Obesity Surgery, focusing on endocrinology, metabolism, and surgical medicine, as well as high-impact medical journals like Lancet. In contrast, the green cluster emphasizes endocrinology, metabolism, nutrition, and multidisciplinary studies, with journals such as the Journal of Clinical Endocrinology and Metabolism, PLOS One, Nutrients, and Diabetes. Lastly, the yellow cluster is dedicated to oncology and geriatrics, featuring journals like Lancet Oncology, Journal of Clinical Oncology, and Journal of Cachexia Sarcopenia and Muscle.

Keywords analysis

The analysis of article keywords provides valuable insights into the primary themes, research directions, and fundamental viewpoints in the field, enabling a comprehensive understanding of research trends and advancements. Table 5 presents the top 20 keywords based on frequency of occurrence and total link strength. The most prevalent keyword, sarcopenic obesity, appears 492 times, surpassing obesity, the second-ranked keyword, by more than twice the frequency (228 times). Additionally, body composition (159 times) and aging (154 times) emerge as significant topics, reflecting the widespread interest and research focus on addressing sarcopenic obesity.

**Table 5:** Ranking of the top 20 major keywords of sarcopenic obesity intervention from 2004 to 2024.

Rank	Keyword	No. of occurrences	Total link strength	Rank	Keyword	No. of occurrences	Total link strength
1	Sarcopenic obesity	492	1117	11	Skeletal muscle	33	87
2	Obesity	228	608	12	Inflammation	30	82
3	Body composition	159	418	13	Metabolic syndrome	29	71
4	Aging	154	421	14	Cancer	28	82
5	Muscle mass	47	146	15	Resistance training	28	77
6	Exercise	46	163	16	Muscle	26	71
7	Nutrition	41	131	17	Muscle strength	26	84
8	Frailty	37	87	18	Diabetes	25	69
9	Body mass index	33	104	19	Cardiovascular disease	24	62
10	Malnutrition	33	84	20	Physical activity	24	75

**Figure 14:**
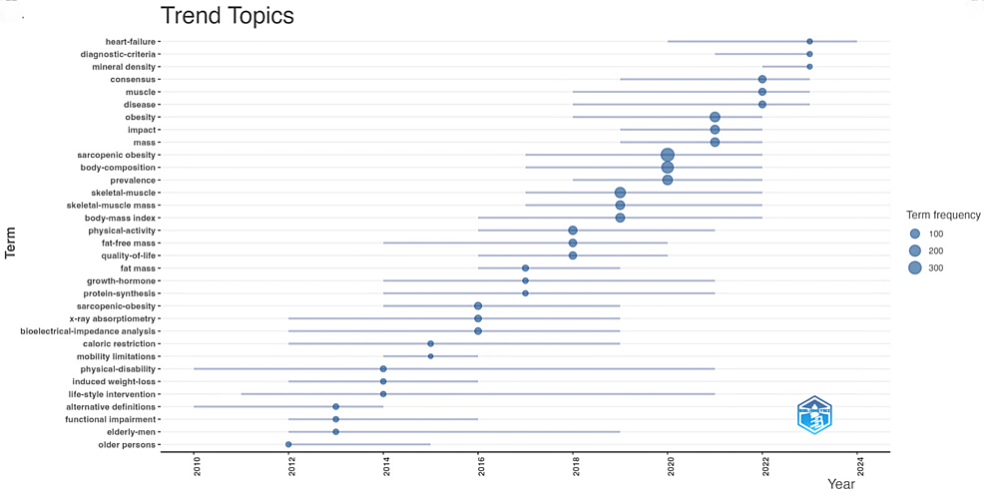
Keywords co-occurrence network mapping and correlation analysis of sarcopenic obesity intervention research from 2004 to 2024. The visualization analysis depicts the duration of keyword popularity over time for the top 30 keywords ranked by frequency from 2012 to 2024. In this visualization, line length represents the duration of popularity, while dot size indicates the frequency of occurrence, organized chronologically. All keyword information data in the figure are sourced from the Web of Science Core Collection (WoSCC) and visualized using VOSviewer.

Figure 14 depicts the fluctuation in keyword frequency from 2010 onwards. The length of the horizontal lines corresponds to the duration of keyword popularity, while the size of the dots reflects the frequency of occurrence. Notably, keywords like sarcopenic obesity, body composition, prevalence, and skeletal muscle mass show higher frequencies. Peak periods of popularity are observed around 2019 and 2020, suggesting significant advancements in addressing sarcopenic obesity. Additionally, heart failure, positioned at the top of the graph, remains a topic of consistent research interest. A detailed examination of this keyword could unveil the latest developments in the field.

**Figure 15:**
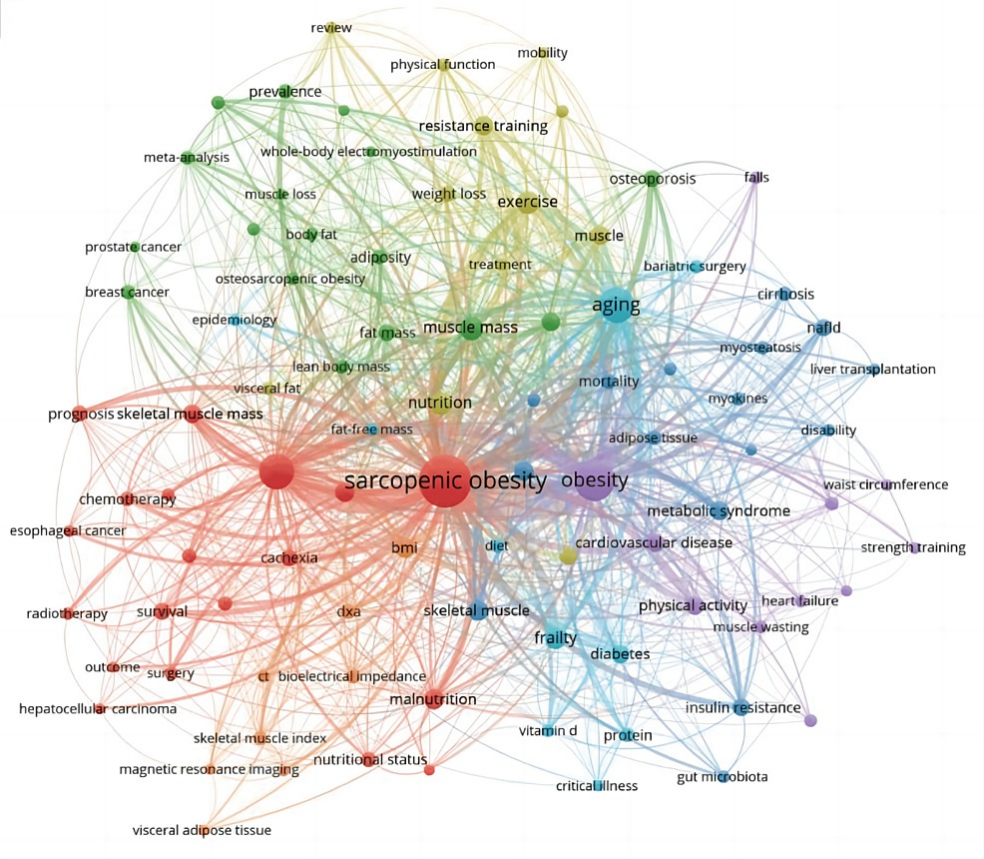
Keywords co-occurrence network mapping and correlation analysis of sarcopenic obesity intervention research from 2004 to 2024. The keyword map illustrates the connections between studied keywords in sarcopenic obesity intervention research. Nodes, categorized by color, represent different clusters of keywords. Node size indicates the frequency of co-occurrence, while connections between nodes depict relationships among keywords. All keyword information data in the figure are sourced from the Web of Science Core Collection (WoSCC) and visualized using VOSviewer.

Figure 15 illustrates the co-occurrence relationships among keywords, providing insight into common themes across different research topics. The central red circle, representing sarcopenic obesity, is associated with terms like skeletal muscle mass, malnutrition, surgery, and prognosis, which predominantly pertain to clinical and surgical aspects. The green cluster above includes terms related to body composition, physiology, and pathology, such as fat mass, muscle mass, lean body mass, and adiposity. The light blue and purple clusters on the right encompass terms like aging, obesity, cardiovascular disease, diabetes, and metabolic syndrome, which are primarily physiological or pathological in nature. The yellow and orange clusters mainly consist of clinical indicators and treatment-related keywords, such as nutrition, exercise, resistance training, DXA, BMI, and CT.

**Figure 16:**
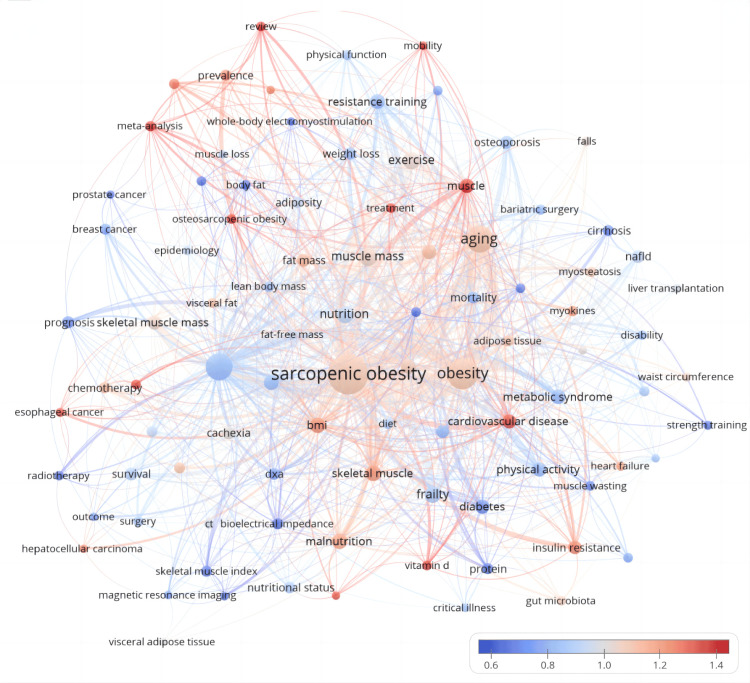
Keywords co-occurrence network mapping and correlation analysis of sarcopenic obesity intervention research from 2004 to 2024. A heat display based on Figure 15 is superimposed on the graph, showing the recent attention level of different keywords by different color shades. All keyword information data in the figure are sourced from the Web of Science Core Collection (WoSCC) and visualized using VOSviewer.

**Figure 17:**
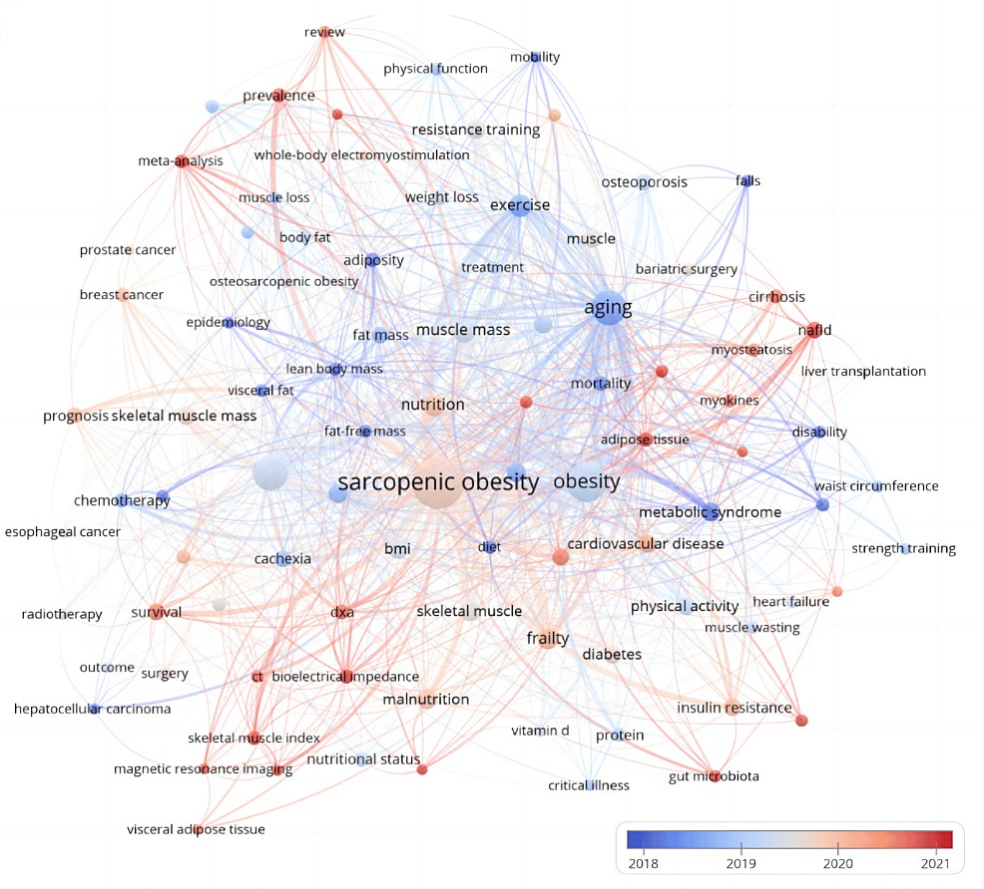
Keywords co-occurrence network mapping and correlation analysis of sarcopenic obesity intervention research from 2004 to 2024. The figure illustrates the relationship between the recent contribution of each keyword to research on sarcopenic obesity intervention and its overall output. Red color indicates an increase in influence and blue color indicates a decrease in attention in the field. The color scale reflects the proportion of keywords over the past five years, highlighting terms that have had a significant impact or reduced participation in this research. All keyword information data in the figure are sourced from the Web of Science Core Collection (WoSCC) and visualized using VOSviewer.

Figures 16, 17, which consider strength and temporal aspects, highlight the prominent keywords such as muscle, treatment, osteosarcopenic obesity, meta-analysis, and review in the field. Recent terms gaining popularity are adipose tissue, Non-alcoholic Fatty Liver Disease Outcome (NAFLD), skeletal muscle index, and meta-analysis, shedding light on recent developments in sarcopenic obesity research. These analyses enhance our comprehension of the changing trends and current emphases in the realm of intervention research for sarcopenic obesity.

**Figure 18:**
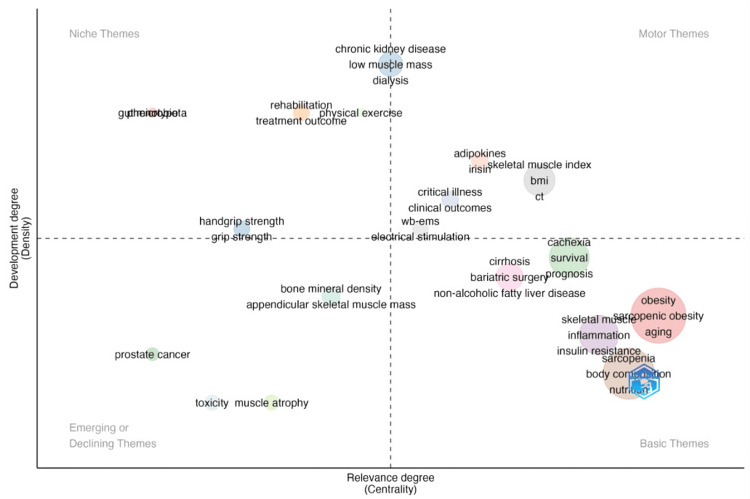
Keywords co-occurrence network mapping and correlation analysis of sarcopenic obesity intervention research from 2004 to 2024. The x-axis represents relevance, while the y-axis indicates development level. Keywords in the upper-right quadrant show the highest relevance and significant development. All keyword information data in the figure are sourced from the Web of Science Core Collection (WoSCC) and visualized using VOSviewer.

Figure 18 succinctly summarizes the discussion trends of important keywords in the field since 2004. The horizontal axis represents the frequency or intensity of keywords, while the vertical axis measures their temporal relevance. Representative keywords are plotted in four quadrants. Keywords in the first quadrant (top-right corner) appeared early and had high intensity, including skeletal muscle index, BMI, CT, adipokines, and irisin. The second quadrant to the left includes keywords that appeared early but received relatively less attention, such as handgrip strength, rehabilitation, and treatment outcome. Terms like prostate cancer, toxicity, muscle atrophy, and bone mineral density in the third quadrant (bottom-left corner) are less relevant and significant in the field. Keywords in the fourth quadrant, which appeared more recently and quickly gained attention in the field, include obesity, aging, skeletal muscle, sarcopenia, cirrhosis, and cachexia.

**Figure 19:**
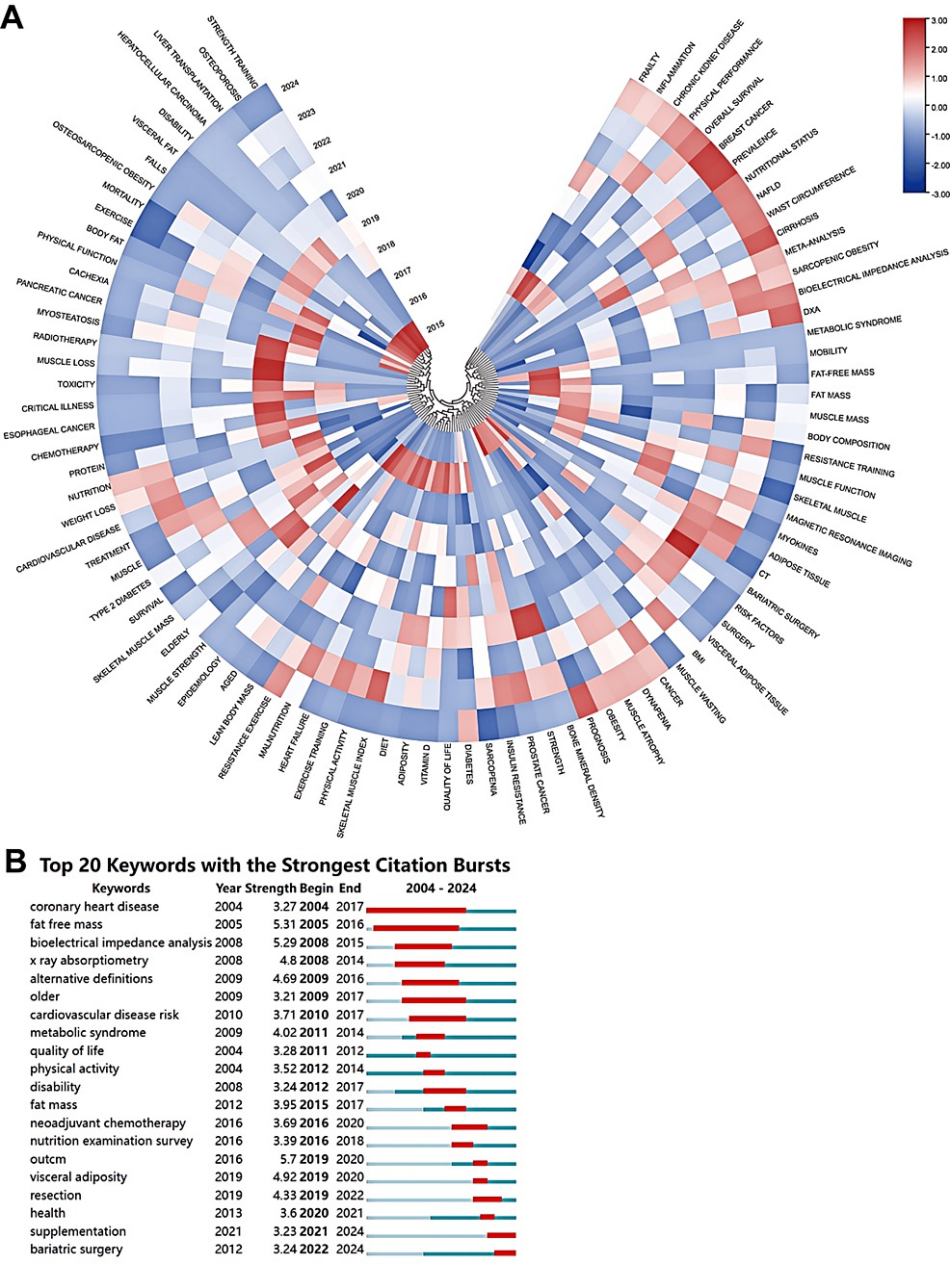
Keyword correlation analysis in the field of sarcopenic obesity intervention from 2004 to 2024. (A) The heatmap displays keyword popularity in sarcopenic obesity intervention research, categorizing keywords into distinct groups based on their popularity within similar timeframes and differentiated by color. (B) The diagram illustrates the 20 primary keywords characterized by pronounced bursts of citations, denoted by red spikes on the timeline. These bursts signify sudden surges in citation counts, signaling pivotal moments of emerging crucial questions or solutions within the field. All keyword information data in the figure are sourced from the Web of Science Core Collection (WoSCC) and visualized using VOSviewer.

Figure 19A presents a comprehensive analysis of the evolution of keyword intensity over time, displayed in a heatmap format. The heatmap illustrates that in 2024, there was a notable focus on terms such as breast cancer, prevalence, cirrhosis, and prognosis. In contrast, keywords like radiotherapy, muscle loss, muscle strength, and liver transplantation were prominent in the field prior to 2019. Figure 19B illustrates the outbreak intensity of the top 20 keywords, demonstrating that keywords with the highest outbreak intensity, such as "outcome" (intensity 5.7), experienced a brief outbreak period from 2019 to 2020. Keywords with similar characteristics, like "visceral adiposity" (4.92) and "resection" (4.33), also had high outbreak intensities. On the other hand, keywords like "coronary heart disease" (3.27) and "fat-free mass" (5.31) emerged early and had a longer outbreak period. It is worth mentioning that "supplementation" (3.23) and "bariatric surgery" (3.24) are still in an outbreak phase, underscoring their ongoing significance in current research on sarcopenic obesity intervention.

Highly cited references analysis

The significance of an article can be gauged by the number of citations it receives, which also reflects its influence in the academic community. Analyzing highly cited articles can provide valuable insights into the current research trends in a particular field. Table 6 outlines key details of the top 15 most cited articles, with the leading article being "Sarcopenia: European consensus on definition and diagnosis" by Cruz-Jentoft AJ and Baeyens JP, published in 2010 in Age and Ageing (8143 citations) [12]. This article holds a prominent position in the field, significantly surpassing the next nine articles in terms of citations. It delves into the definition and diagnosis of sarcopenia, examining its associations with cachexia, frailty, and sarcopenic obesity. Expanding on the original European Working Group on Sarcopenia in Older People (EWGSOP) definition, the article proposes a novel approach to defining sarcopenia in older adults, incorporating factors like gait speed, grip strength, and muscle mass. The second most cited article, with 2187 citations, is "Prevalence and clinical implications of sarcopenic obesity in patients with solid tumors of the respiratory and gastrointestinal tracts: a population-based study" by Prado et al. published in 2008 in Lancet Oncology [13]. This study, which surveyed 2115 patients with respiratory or gastrointestinal solid tumors, examines the prevalence and clinical impact of sarcopenic obesity. It highlights how variations in fat-free mass can affect the distribution of chemotherapy per unit body-surface area. Another significant article, "European Society for Clinical Nutrition and Metabolism (ESPEN) guidelines on definitions and terminology of clinical nutrition," was published in 2017 by Cederholm et al. in Clinical Nutrition [14]. This publication, cited 1285 times, aimed to establish standardized terms in the field of clinical nutrition by achieving consensus among clinical scientists. This agreement on terminology is essential for facilitating future global consensus efforts and revisions to classification systems.

**Table 6:** Ranking of the top 15 major highly cited references of sarcopenic obesity intervention from 2004 to 2024.

Rank	Author	Article title	Journal	No. of citations	Year	Category	DOI
1	Cruz-Jentoft et al. [12]	Sarcopenia: European consensus on definition and diagnosis	Age and Ageing	8143	2010	Article	10.1093/ageing/afq034
2	Prado et al. [13]	Prevalence and clinical implications of sarcopenic obesity in patients with solid tumours of the respiratory and gastrointestinal tracts: a population-based study	Lancet Oncology	2187	2008	Article	10.1016/S1470-2045(08)70153-0
3	Cederholm et al. [14]	ESPEN guidelines on definitions and terminology of clinical nutrition	Clinical Nutrition	1285	2017	Article	10.1016/j.clnu.2016.09.004
4	Kalyani et al. [15]	Age-related and disease-related muscle loss: the effect of diabetes, obesity, and other diseases	Lancet Diabetes and Endocrinology	670	2014	Review	10.1016/S2213-8587(14)70034-8
5	Batsis and Villareal [16]	Sarcopenic obesity in older adults: aetiology, epidemiology and treatment strategies	Nature Reviews Endocrinology	648	2018	Review	10.1038/s41574-018-0062-9
6	Zamboni et al. [17]	Sarcopenic obesity: a new category of obesity in the elderly	Nutrition, Metabolism and Cardiovascular Diseases	589	2008	Review	10.1016/j.numecd.2007.10.002
7	Zamboni et al. [18]	Health consequences of obesity in the elderly: a review of four unresolved questions	International Journal of Obesity	450	2005	Review	10.1038/sj.ijo.0803005
8	Srikanthan et al. [19]	Sarcopenia exacerbates obesity-associated insulin resistance and dysglycemia: findings from the National Health and Nutrition Examination Survey III	PLOS One	412	2010	Article	10.1371/journal.pone.0010805
9	Walston [20]	Sarcopenia in older adults	Current Opinion in Rheumatology	399	2012	Review	10.1097/BOR.0b013e328358d59b
10	Prado et al. [21]	Sarcopenic obesity: a critical appraisal of the current evidence	Clinical Nutrition	381	2012	Review	10.1016/j.clnu.2012.06.010
11	Prado and Heymsfield [22]	Lean tissue imaging: a new era for nutritional assessment and intervention	Journal of Parenteral and Enteral Nutrition	372	2014	Article	10.1177/0148607114550189
12	Shah and Braverman [23]	Measuring adiposity in patients: the utility of body mass index (BMI), percent body fat, and leptin	PLOS One	371	2012	Article	10.1371/journal.pone.0033308
13	Villareal et al. [24]	Aerobic or resistance exercise, or both, in dieting obese older adults	New England Journal of Medicine	355	2017	Article	10.1056/NEJMoa1616338
14	Cleasby et al. [25]	Insulin resistance and sarcopenia: mechanistic links between common co-morbidities	Journal of Endocrinology	327	2016	Review	10.1530/JOE-15-0533
15	Cruz-Jentoft et al. [26]	Nutrition, frailty, and sarcopenia	Aging Clinical and Experimental Research	302	2017	Review	10.1007/s40520-016-0709-0

Sarcopenic obesity (SO) refers to the coexistence of obesity and sarcopenia, characterized by a decline in skeletal muscle mass, strength, and function as well as an increase in body fat, commonly observed with aging. Individuals, regardless of age, who are obese are at a higher risk of developing sarcopenia due to adipose tissue-related metabolic disorders such as oxidative stress, inflammation, and insulin resistance. This negative impact of obesity on muscle mass and function is further exacerbated by the presence of chronic non-communicable diseases. A sedentary lifestyle contributes to the development of SO, forming a cycle of cause and effect. Weight loss interventions aimed at reducing excess fat can result in the loss of skeletal muscle mass, particularly evident post-bariatric surgery without proper nutritional guidance. Conversely, sarcopenia can lead to increased fat accumulation due to a decrease in total energy expenditure, creating a synergistic relationship between obesity and sarcopenia [27].

Among the 65 countries and regions contributing to the literature on interventions for sarcopenic obesity, the United States has the highest publication output and citations, along with significant international collaboration. Italy, France, the United Kingdom, and Germany have also made noteworthy contributions. The United States and Europe are recognized as primary academic centers for research on sarcopenic obesity intervention, marked by robust academic exchanges. Additionally, the publication volume from China, South Korea, and Japan is rapidly increasing, likely due to their aging populations. Leading authors in this field include Boirie Y from Université Clermont Auvergne in France and Prado CM from the University of Alberta in Canada. Noteworthy for citation count are Cruz-Jentoft AJ from Hospital Universitario Ramon y Cajal in Spain and Prado CM from the University of Alberta. The University of Alberta leads in the number of publications, while the University of Verona in Italy leads in citation frequency. Strong collaboration is observed between Uppsala University in Sweden, the University of Trieste in Italy, the University of Nottingham in the United Kingdom, Johns Hopkins University in the United States, and Sapienza University of Rome in this research field. Journals with higher publication volumes in sarcopenic obesity intervention include Nutrients, Clinical Nutrition, and Journal of Cachexia Sarcopenia and Muscle. Notably, all top 10 journals in terms of citations are in Q2 and above, with seven in Q1.

Among the top 20 keywords, those most relevant to interventions for sarcopenic obesity include exercise, nutrition, resistance training, physical activity, and muscle strength. It is recommended that, at this point, exercise - particularly enhancing muscle strength through resistance training - and nutritional support are the primary approaches for addressing obese sarcopenia. The effects of dietary intervention in managing sarcopenic obesity are a subject of debate, with previous studies lacking sufficient behavior change techniques crucial for successful dietary interventions. In a pilot randomized controlled trial, 60 older adults (≥60 years old) with sarcopenic obesity were divided into an experimental group (n=30) receiving a 15-week dietary intervention with behavior change techniques based on the Health Action Process Approach model, and a control group (n=30) receiving standard health talks. Semi-structured interviews with 21 participants from the experimental group revealed insights into the barriers and facilitators of dietary behavior changes post-intervention. The study demonstrated the feasibility of the dietary behavior change intervention, as evidenced by a satisfactory recruitment rate (57.14%) and a high retention rate (83.33%). Compared to the control group, the experimental group showed significant reductions in body weight (p=0.027, d=1.22) and improvements in dietary quality (p<0.001, d=1.31). Positive changes in handgrip strength (from 15.37±1.08 kg to 18.21±1.68 kg), waist circumference (from 99.28±1.32 cm to 98.42±1.39 cm), and gait speed (from 0.91±0.02 m/s to 0.99±0.03 m/s) were observed exclusively in the experimental group. However, the skeletal muscle mass index decreased in the experimental group [28].

A meta-analysis conducted by Eglseer et al. included studies of community-dwelling individuals aged between 50 and 70 years with sarcopenic obesity who received nutritional or exercise interventions for a duration of at least eight weeks. The primary endpoint examined was body composition, while secondary endpoints included body mass index, muscle strength, and physical function. The meta-analysis focused on the effects of "resistance training" and "training (resistance or aerobic)" in combination with "added protein" compared to "no intervention" or "training alone." The findings revealed that resistance training resulted in a significant reduction in body fat (-1.53%; 95% CI: -2.91 to -0.15), an increase in muscle mass (2.72%; 95% CI: 1.23-4.22), improved muscle strength (4.42 kg; 95% CI: 2.44-6.04), and a slight enhancement in gait speed (0.17 m/s; 95% CI: 0.01-0.34). Additionally, the combination of protein with exercise interventions led to a significant reduction in fat mass (-0.80 kg; 95% CI: -1.32 to -0.28). Although some individual studies on dietary or food supplement interventions showed positive effects on body composition, the overall conclusion of the study was that resistance training is an effective treatment for individuals with sarcopenic obesity in the retirement age group, and combining increased protein intake with exercise may further enhance reductions in fat mass [29]. Another umbrella review of meta‑analyses of randomized controlled trials conducted by Reiter et al. systematically searched for meta-analyses of RCTs on the treatment and prevention of sarcopenic obesity (SO) between 2018 and 2022 in PubMed, Embase, and CENTRAL. The review focused on primary endpoints related to SO, such as body fat percentage, skeletal muscle mass index (SMMI), gait speed, leg strength, and grip strength. Methodological quality was assessed using A Measurement Tool to Assess systematic Reviews (AMSTAR), and the certainty of evidence was evaluated using Grading of Recommendations Assessment, Development, and Evaluation (GRADE). The umbrella review included four systematic reviews with participant numbers ranging from 30 to 225, examining various exercise, nutrition, and combined interventions. The findings indicated that resistance training was the most commonly studied intervention, showing improvements in gait speed (0.14 m/s to 0.17 m/s) and lower leg strength (9.97 kg). However, interventions such as resistance, aerobic, mixed exercise, and hypocaloric diet combined with protein supplementation did not significantly impact outcomes for individuals with SO compared to no intervention. Due to the limited number of primary studies, the certainty of evidence ranged from moderate to very low. Despite this uncertainty, the study suggested that resistance training could be a beneficial intervention for individuals with SO, particularly for enhancing muscle function [30].

Analyzing the top 15 most cited documents provides valuable insights into the evolution and comprehensive landscape of intervention strategies for sarcopenic obesity from 2004 to the present. Notable among these documents are the following three publications in prestigious journals: "Aerobic or resistance exercise, or both, in dieting obese older adults" by Villareal et al. in the New England Journal of Medicine [24], "Sarcopenic obesity in older adults: etiology, epidemiology and treatment strategies" in Nature Reviews Endocrinology by Batsis and Villareal [16], and the study "Age-related and disease-related muscle loss: the effect of diabetes, obesity, and other diseases" authored by Kalyani et al. published in Lancet Diabetes and Endocrinology [15]. Batsis and Villareal discussed the importance of lifestyle interventions, such as calorie restriction and physical activity, in treating sarcopenic obesity in older adults [16]. They highlighted that dietary strategies, including calorie restriction and protein supplementation, are crucial. The article emphasized the significance of adequate protein intake to combat weight loss-induced sarcopenia in individuals with sarcopenic obesity. It also stressed the need for careful medical monitoring and dietary planning, particularly when optimizing protein intake while limiting calorie restriction, which should ideally be overseen by a registered dietitian with expertise in this field. Recognizing the challenges of calorie limitation, the authors suggested the necessity of alternative approaches to enhance muscle mass and strength. Additionally, individualized exercise treatment tailored to patients with sarcopenic obesity was recommended due to associated medical comorbidities and disabilities. Aerobic activities were advised to target around 65% of peak heart rate, gradually increasing to 70-85% over the exercise duration. Resistance activities should involve one to two sets of eight to 12 repetitions at approximately 65% of one repetition maximum, with a goal of progressing to two to three sets at 75% of one repetition maximum over time. These exercises were deemed beneficial even for frail, older adults [16,24,31].

While the most recent article among the top 15 most cited was published in 2018, it is crucial to acknowledge the continued significance of relevant articles published after this year. Several important studies referenced in our work were published post-2018 [28-30]. Furthermore, Figures 15-18 highlight the keyword "bariatric surgery." An article by Rodrigues et al. published in May 2024, investigated the impact of bariatric surgery on sarcopenic obesity outcomes [32]. In this single-center study of 140 patients who underwent Roux-en-Y gastric bypass or sleeve gastrectomy between November 2019 and December 2022, participants were categorized into tertiles based on sarcopenic obesity (SO) diagnosis and severity. Group 1 included patients with the most severe SO, group 2 had intermediate severity, and group 3 had the least severe or no SO, as per the consensus by ESPEN and EASO in 2022. Clinical and biochemical parameters were evaluated before and 12 months after bariatric surgery (BS), with body composition assessed using bone density scans. Linear regression analysis considered surgery type and baseline body mass index (BMI). The study revealed that SO prevalence in the overall sample decreased from 89.3% before BS to 2.9% after BS. Group 1 had higher body fat mass and lower total skeletal muscle mass at baseline compared to groups 2 and 3. One year post-BS, group 1 showed more weight loss, BMI reduction, and fat mass loss, but no significant difference in total skeletal muscle mass reduction. Remission rates for comorbidities were substantial across all groups, with more pronounced improvements in group 1 for type 2 diabetes mellitus, hypertension, and dyslipidemia. Post-BS, there was a notable increase in engagement in physical exercise among patients. The study indicates that despite concerns about malabsorptive mechanisms potentially worsening muscle loss, patients with the most severe obesity undergoing bariatric surgery lost more fat mass while experiencing the smallest reduction in total skeletal muscle mass. Remission rates for comorbidities following bariatric surgery were notable among all groups.

Despite employing a rigorous methodological approach in this bibliometric study to offer a comprehensive overview of research on interventions for sarcopenic obesity from 2004 to the present, there are certain limitations to consider. Firstly, the heavy reliance on the WoSCC database, known for its extensive literary resources and reliable information, may result in an incomplete literature search. To improve coverage, incorporating additional databases like PubMed and Scopus would be advantageous. Secondly, the study focuses solely on English-language literature, potentially overlooking valuable non-English-language sources. Thirdly, the bibliometric analysis does not evaluate the quality of the literature retrieved. Lastly, the study restricts its scope to literature published between 2004 and mid-June 2024, suggesting that researchers interested in the field should also explore newly published research post-mid June 2024.

## Conclusions

As the aging population accelerates and issues such as fast-paced, high-stress, and sedentary lifestyles in modern society become more prominent, sarcopenic obesity has garnered increased attention. Currently, the most abundant research evidence for improving muscle strength in sarcopenic obesity patients is based on resistance training. Protein supplementation in conjunction with resistance exercise has shown promise in further reducing fat mass in these patients. Clinical studies have also indicated that dietary behavior change interventions can be beneficial for individuals with sarcopenic obesity. However, the population of sarcopenic obesity patients is highly diverse. Therefore, individualized and comprehensive intervention plans should be tailored considering factors such as age, multiple chronic conditions, physical functional status, life expectancy, and other relevant aspects. It is crucial to ensure that the intervention plan devised is sustainable in the long term for the patient. Interventional research on sarcopenic obesity spans across various disciplines including clinical nutrition, endocrinology and metabolism, geriatric medicine, and sports medicine. Moving forward, it is essential to enhance cooperation among countries/regions and academic institutions in this field and promote multidisciplinary collaboration. By working together, conducting high-quality clinical studies can lead to the exploration of more effective and feasible interventions for individuals with sarcopenic obesity.
